# Ginsenosides improve reproductive capability of aged female *Drosophila* through mechanism dependent on ecdysteroid receptor (ECR) and steroid signaling pathway

**DOI:** 10.3389/fendo.2022.964069

**Published:** 2022-08-09

**Authors:** Baoyu Fu, Rui Ma, Fangbing Liu, Xuenan Chen, Xiaoyu Teng, Pengdi Yang, Jianzeng Liu, Daqing Zhao, Liwei Sun

**Affiliations:** ^1^ Research Center of Traditional Chinese Medicine, Affiliated Hospital, Changchun University of Chinese Medicine, Changchun, China; ^2^ Jilin Ginseng Academy, Changchun University of Chinese Medicine, Changchun, China; ^3^ College of Science, Beihua University, Jilin, China; ^4^ Key Laboratory of Active Substances and Biological Mechanisms of Ginseng Efficacy, Ministry of Education, Changchun University of Chinese Medicine, Changchun, China

**Keywords:** ginsenosides, aged female *Drosophila*, reproductive capacity, *ECR*, steroid signaling pathway

## Abstract

Aging ovaries caused diminished fertility and depleted steroid hormone level. Ginsenosides, the active ingredient in ginseng, had estrogen-like hormonal effects. Although ginsenosides were well known for their ability to alleviate many age-related degenerative diseases, the effect of ginsenosides on the decline in reproductive capability caused by aging, as well as the mechanism, are unknown. We found that ginsenosides improved the quantity and quality of the offspring, prolonged life and restored muscle ability in aged female *Drosophila.* In addition, ginsenosides inhibited ovarian atrophy and maintained steroid hormone 20-Hydroxyecdysone (20E) and juvenile-preserving hormone (JH)) levels. Ginsenosides activated ecdysteroid receptor (ECR) and increased the expression of the early transcription genes E74 and Broad (Br), which triggered steroid signaling pathway. Meanwhile, ginsenosides promoted JH biosynthesis by increasing the expression of Hydroxyl-methylglutaryl-CoA reductase (HMGR) and juvenile hormone acid O-methyltransferase (JHAMT). Subsequently, JH was bound to Methoprene Tolerant (Met) and activated the transcription of the responsive gene Kruppel Homolog 1 (Kr-h1), which coordinated with 20E signaling to promote the reproduction of aged female *Drosophila*. The reproductive capacity and steroid hormone levels were not improved and the steroid signaling pathway was not activated in ginsenoside-treated ECR knockout *Drosophila*. This suggested that ginsenosides played a role dependent on targeted ECR. Furthermore, 17 kinds of ginsenoside monomers were identified from the total ginsenosides. Among them, Rg1, Re and Rb1 improved the reproductive capacity and steroid hormone levels of aged female *Drosophila*, which has similar effects to the total ginsenoside. These results indicated that ginsenosides could enhance the reproductive capacity of aged female *Drosophila* by activating steroid signals dependent on nuclear receptor ECR. In addition, ginsenoside monomers Rg1, Rb1 and Re are the main active components of total ginsenosides to improve reproductive ability. This will provide strong evidence that ginsenosides had the potential to alleviate age-induced reproductive degradation.

## Graphical Abstract

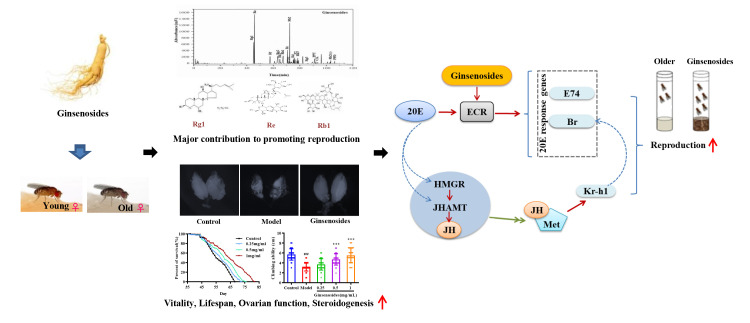

## Highlights

1. Ginsenosides enhanced the reproductive capacity of aged female *Drosophila*.

2. Ginsenosides repaired ovarian size and restored steroid hormone secretion.

3. Ginsenosides activated steroid signals dependent on nuclear receptor ECR.

4. Ginsenosides play an estrogen-like role to alleviate age-induced ovarian dysfunction.

5. Rg1, Rb1 and Re might play a major contribution to promoting reproduction.

## Introduction

During the past 150 years, the average lifespan of human beings has increased from 45 to 85 years, while the reproductive lifespan of women has remained constant at 50-52 years (1). The social phenomenon of late marriage and late childbearing of women has been formed with the extension of human life span and the change of life style. Therefore, it is urgent to prolong reproductive life and improve fertility. The ovaries, known as the pacemakers of reproductive aging, are the endocrine organs that control female reproduction. According to the report, women typically begin to lose ovarian function at the age of 35, manifested by reduced reproductive function and steroid deficiency, which negatively affects quality of life (2, 3). At present, most clinical treatments are based on exogenous hormone therapy, which can alleviate reproductive decline. However, when such treatments are administered long-term, they can trigger side effects such as hypertension and edema, while also increasing risks of incidence of gynecologic tumors associated with diseases such as breast and endometrial cancers (4–6). Therefore, a hormone substitute for estrogen is urgently needed that can preserve reproductive capacity without adverse side-effects.

Ginseng (*Panax ginseng* C. A. Meyer) is a traditional herb, which has been widely used to address aging, healthy life expectancy and ovarian dysfunction (7, 8). Ginsenosides is a steroid compound, regarded as the active ingredient with high content in ginseng, which has a variety of pharmacological activities such as relieving body fatigue, enhancing immunity, improving sleep and protecting nerves (9). Multiple pharmacological researches have confirmed that ginsenosides can enhance D-galactose-induced estrous cycle in ovarian senescent mice and help repair oxidative stress-induced damage to ovarian reproductive function (10). Ginsenosides is able to accelerate sexual maturation and promote ovulation in dinbutyl phthalate-induced reproductive impairment in mice (11). It also enhances egg production in cholesterol-deprived Caenorhabditis elegans (12). Ginsenosides can increase the steroid hormone output of adrenocortical cells in Y1 mice to help alleviate ovarian endocrine disorders (13). Ginsenoside Rg1 has been found to have estrogen-like effects *in vivo* by binding to estrogen receptors (14, 15). All of this implied that ginsenosides may play an estrogen-like role in improving ovarian aging due to their similar structure to steroids.

In old age, estrogen production decreases with loss of ovarian function, which leads to age-related diseases such as heart disease, osteoporosis, and Alzheimer’s disease (16, 17). Estrogen is a steroid hormone that promotes the development of the reproductive system and enhances sexual behavior. Studies had shown that steroid hormone levels in older women were restored to repair damaged ovarian function (18). The steroid hormone most similar to estrogen in *Drosophila* is 20-Hydroxyecdysone (20E) (19). It had been reported that 20E binds to its receptor ecdysteroid receptor (ECR) to induce expression of E74 and Broad (Br) transcription factors that regulate female accessory gland development and ovum formation (7, 20). Meanwhile, 20E indirectly influences juvenile-preserving hormone (JH) synthesis that controls sexual development, induces sexual priming, and promotes oocyte maturation (21, 22). However, there were no reports on the regulation of fertility by ginsenosides through steroidal signaling.

In recent years, *Drosophila* has served as a classic *in vivo* model for use in studying aging and reproduction (23), since *Drosophila* reproductive biology resembles that of mammals (24, 25). As compared to other animal models, *Drosophila* has a simple ovarian structure, short reproductive cycle, and easy gene editing (26). In this study, we investigated effects of ginsenosides on reproductive capacity, life energy and steroid hormone levels in natural aging female *Drosophila*. In addition, the regulation of steroidal signals on reproductive ability was discussed. The main contributing components of ginseng with improved reproductive ability were further identified and screened. This is of great significance for improving female reproductive ability and prolonging reproductive life.

## Materials and methods

### Materials

Ginsenosides from *Panax* ginseng roots, ginsenoside Rg1, Re, Rf, Rg2, Rh1, Rb1, Rc, F1, Rb2, Rb3, Rd, F2, Rk3, Rg3, PPT, CK, Rh2(s), and PPD, each of > 98% purity, were purchased from Shanghai Yuanye Bio-Technology Co., Ltd. (Shanghai, China).

### *Drosophila* source and maintenance

*Drosophila* were obtained from the *Drosophila* research repository of the Experimental Center of the Affiliated Hospital of Changchun University of Chinese Medicine. ECR knock-down mutant *Drosophila* were obtained from the Core Facility of *Drosophila* Resource and Technology, CEMCS, CAS. All the study protocols were approved by the Ethics Committee of Changchun University of Chinese Medicine of TCM (No: 2021349) and conducted according to the guidelines of the Ethics Committee of Changchun University of Chinese Medicine of TCM. *Drosophila* were reared on standard cornmeal-sugar-yeast agar medium that contained 8% sugar (Tianjin Xinbote, China), 0.5% Oxoid LP0021 yeast extract (Oxoid, United Kingdom), 0.5% propionic acid (Sinopharm., China), and 2% agar (Biofroxx, Germany). Ginsenosides were added in medium to final concentrations of 0.25, 0.5 and 1 mg/mL for further experiments. All *Drosophila* were maintained and reared under conditions of 25°C with 60% humidity and a 12-h light/12-h dark cycle.

### Assay to assess reproduction

The virgin female *Drosophila* aged 7 and 35 days were collected under CO_2_ anesthesia and assigned to randomly assigned to five subgroups that included the control group (7 days), model group (35 days), and three ginsenosides treated groups of 35-day-old female *Drosophila*. Female *Drosophila* in the untreated control group and untreated model group consumed standard medium, while 35-day-old female *Drosophila* in the other three groups received medium containing ginsenosides at concentrations of 0.25, 0.5 and 1 mg/mL. After 7 days, all groups were transferred to tubes containing standard medium. Next, a total of 100 mated female *Drosophila* in one tube each were transferred to fresh tubes containing standard medium, where they remained for 48 h while they laid eggs. Number of eggs, number of pupae, and pupation rate for each tube of female *Drosophila* were recorded and analyzed to determine effects of ginsenosides on female *Drosophila* development (27).

### Lifespan assay

Female *Drosophila* (35-day-old) were divided into four groups (100 flies/group): control group (35 days) female *Drosophila* were fed standard medium, while the other three groups (35 days female *Drosophila*) received medium containing ginsenosides concentrations of 0.25, 0.5 and 1 mg/mL, respectively. Every three days, female *Drosophila* in all groups were transferred to tubes containing fresh culture medium then mortality rates were recorded. Survivorships were scored regularly and eliminated the unnatural death flies, and death flies were removed out of the cage when possible. Lifespan curves, mean lifespan (average value of all values), and maximum lifespan (average values of the last 20 dead flies) were calculated for each group of female *Drosophila* (28).

### Climbing assay

Female *Drosophila* aged 7 days and 35 days were collected under CO_2_ anesthesia and assigned to randomly assigned to five subgroups: a 7-day-old untreated control group, a 35-day-old untreated model group, and three 35-day-old *Drosophila* groups treated with different concentrations of ginsenosides (0.25, 0.5, and 1 mg/mL). *Drosophila* in the untreated control group (7 days) and the untreated model group (35 days) ate standard food, while *Drosophila* in the other three groups ate food containing ginsenosides. Prior to climbing experiments, female *Drosophila* received food with or without ginsenosides for 7 days. Exercise ability was evaluated using a negative axis test with maximum crawl path restricted to 12 cm in length. After a 10-min acclimation period in the test tube, female *Drosophila* were knocked to the bottom of the tube every 1 min then this procedure was repeated 5 times followed by counting of crawling heights of female *Drosophila*, with assays repeated at least 3 times (29).

### Ovarian measurements

To explore the effect of ginsenosides on ovarian morphology, ovaries were dissected in anatomical buffer at room temperature. Briefly, 5-10 adult flies per group were anesthetized with CO_2_, and ovaries were isolated with the aid of forceps. The length and diameter of individual ovaries were measured under a stereomicroscope (SMZ1000 with Digital Sight DS-U3, Nikon, Japan).

### Measurement of steroid hormone content

After female *Drosophila* were pretreated with ginsenosides for 7 days, female *Drosophila* ovaries were collected under CO_2_ anesthesia, and 100 ul of RIPA lysate per 10 ovaries was added for tissue crushing. After crushing, the supernatant was centrifuged at 12,000 rpm for 5 minutes, and the supernatant was taken out for subsequent experiments. Contents of 20E and JH were measured using enzyme-linked immunosorbent assays (Sino Best Biological Technology Co., Ltd., Shanghai, China) based on methods as described by the manufacturer.

### Reverse transcription-quantitative PCR analysis (RT-qPCR)

The female *Drosophila* ovaries pretreated with ginsenosides for 7 days were weighed and frozen in liquid nitrogen. Total RNA was extracted using a Magnetic Tissue/Cell/Blood Total RNA Kit (Invitrogen, Carlsbad, CA, USA). Next, 1 µg of RNA was reverse transcribed into cDNA using the script cDNA synthesis kit (Bio-Rad, CA, United States). Subsequently, RT-qPCR was performed in a Bio-Rad CFX96 system using the SYBR Premix Ex Taq Kit (Takara Biomedical Technology Co., Ltd, Beijing, China). Set as 95°C for 5 min, 95°C for 15 s, 60°C for 30 s, and 72°C for 30 s for 40 cycles. Steroid signal pathway genes were selected, with the β-actin gene used as the internal control. Primer sequences are shown in **Table 1**. Comparisons among groups were made based on gene expression levels calculated using the threshold cycle (Ct) method. Gene expression levels in all groups were expressed as ratios of values for each group to corresponding values obtained for the control group (30).

**Table 1:** Primer sequences used for quantification of gene expression.

Genes	Forward Primer sequence	Reverse Primer sequence
Met	GCCAGTAAGCATTACCAGCGAGAG	TGGAGGCAGTAGAACGAGGTGAC
JHAMT	GCCAGTAAGCATTACCAGCGAGAG	TGGAGGCAGTAGAACGAGGTGAC
ECR	CGCTGGACTCGCACGACTATTG	CGCTCTGCTGCTGCTGACTTAG
Br	GCAACAACAGCAGCAGCAACAG	GTGTGGTGGTGGGCGTATTGG
E74	CGTCGGAGAGGAGAGTGGAGTG	TTGGTGTGCGTGTGCTGTGTAC
HMGR	GTCCCATAAAGCCTCCACGC	TTGTGGAGTGGGCAGTGAGT
Kr-h1	TGTGGCATGACCTTTGGCAG	CTCCAGAGGCGCCATTAAGC
β-actin	TTGTCTGGGCAAGAGGATCAG	ACCACTCGCACTTGCACTTTC

### Western blot analysis

Total protein was extracted from ovaries with lysis buffer (Beyotime, Haimen, China) and Bullet Blender (NY, USA). Protein concentration was determined using bicinchoninic acid (BCA) protein assay reagent. Glyceraldehyde 3-phosphate dehydrogenase (GAPDH, BM1623; Boster, Beijing, China) was used as the loading control. Samples were separated by 12% SDS-PAGE (Bio-Rad, Hercules, CA, USA) and transferred to polyvinylidene difluoride membranes (Millipore, USA). After blocking in 5% non-fat milk, membranes were immunoblotted with the following antibodies: ECR, Br (both from Developmental Studies Hybridoma Bank, USA). Kr-h1 (Santa Cruz Biotechnology, Santa Cruz, USA). Next, the membranes were incubated with appropriate secondary antibodies (Li-Cor, Lincoln, NE, USA) for 1 h at room temperature (31). Finally, chemiluminescence-based visualization and analysis of protein bands were conducted using a FluorChem imaging system (ProteinSimple, San Jose, CA, USA).

### Identify and quantify ginsenosides by HPLC

Ginsenosides composition was analyzed and quantified *via* HPLC analysis using a LC-2030C Plus system equipped with a C18 column (5 µm, 4.6 × 250 mm; Kromasil, Sweden), with ultra-violet (UV) detection conducted at 203 nm. A stepwise gradient elution method was employed using solvent A (water) and solvent B (acetonitrile) that were maintained at 30°C. The flow rate was 1 mL/min and sample injection volume was 10 µL (32, 33).

### Statistical analysis

All test data were expressed as means ± standard error of mean (SEM) based on data obtained for three replicate samples. Using D’Agostino-Pearson omnibus K2 testing, and Shapiro-Wilk-test to assess normality, and data are in accordance with normal distribution. After checking the normality and homogeneity, all the data with normal distribution and homogenous variances were analyzed by One-way ANOVA. Statistical analysis was performed using GraphPad Prism 6 (Version No. 6, GraphPad Software, La Jolla, CA, USA). Statistical significance was established using one-way ANOVA followed by Dunnett’s t-test except for survivorship results. Survivorship values among groups were compared and tested for significance using a log rank test. Statistical significance was set to ∗ *p* < 0.05, ∗∗ *p* < 0.01, and ∗∗∗ *p* < 0.001.

## Results

### Effects of ginsenosides on reproduction of aged female *Drosophila*

**Figure 1:**
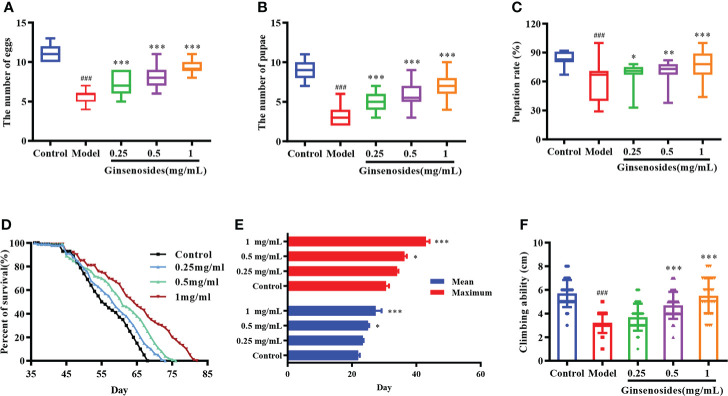
Effects of ginsenosides on *Drosophila* reproduction and aging. **(A)** The number of eggs; **(B)** The number of pupae; **(C)** Pupation rate; **(D)** Percent of survival; **(E)** Mean and maximum lifespans; **(F)** Climbing ability. ^###^ *p*<0.001 compared with control (7-day-old female *Drosophila*); *p<0.05, **p<0.01, ***p<0.001 compared with model (35-day-old female *Drosophila*).

Ovarian function declines as a woman ages, resulting in reduced reproductive capacity. In order to determine ginsenosides effects on reproductive capacity of aged female *Drosophila*, 35-day-old female *Drosophila* were fed ginsenosides at different concentrations. For model group (untreated 35-day-old female *Drosophila*), numbers of eggs, pupae, and pupation rates were significantly lower than corresponding results obtained for control group (7-day-old untreated female *Drosophila*) (**Figure 1**). In contrast, the numbers of eggs of older female *Drosophila* fed with 0.25 mg/mL, 0.5 mg/mL and 1 mg/mL ginsenosides were increased significantly, and 1 mg/mL ginsenosides increased the number of eggs 1.7-fold. The number and rate of pupae of older female *Drosophila* were increased with the ginsenoside concentration, with the highest concentration increasing by 2-fold and 1.2-fold. (**Figures 1A–C**). These results suggested that ginsenosides could promote increased reproductive capacity when fed to elderly female *Drosophila*.

### Effects of ginsenosides on lifespan and climbing ability

The decline of ovarian function could accelerate aging, leading to a shortened life span and reduced body function. In order to observe the effects of ginsenosides on the life span of aged female *Drosophila*, 35-day-old female *Drosophila* were fed ginsenosides at different concentrations (0.25 mg/mL, 0.5 mg/mL, and 1 mg/mL). The life span of female *Drosophila* was extended with the increase ginsenosides concentration (**Figure 1D**). In addition, ginsenosides also extended the average and maximum life span of female *Drosophila* in a concentration dependent manner (**Figure 1E**). Climbing ability can be used to evaluate the aging level of *Drosophila* vitality. The climbing height of 35-day-old *Drosophila* was lower than that of 7-day-old *Drosophila*. Ginsenosides increased the climbing ability of aged female *Drosophila*. The climbing height of aged female *Drosophila* fed 1 mg/mL ginsenoside increased by 71% compared with that without ginsenosides (**Figure 1F**). Senile female *Drosophila* treated with ginsenosides alleviated aging and restored youthful vigor.

### Effects of ginsenosides on ovarian function

**Figure 2:**
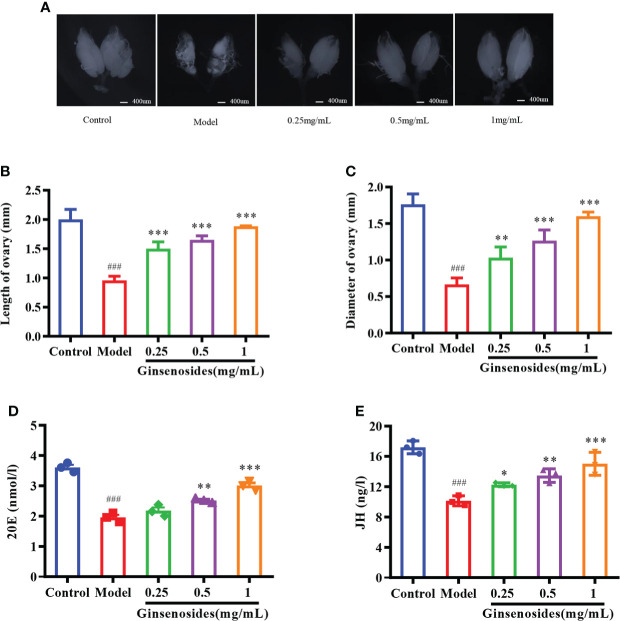
Effect of ginsenosides on ovarian function in *Drosophila*. **(A)** Ovary size; **(B)** Length of ovary; **(C)** Diameter of ovary; **(D)** 20E content; **(E)** JH content. *p<0.05, **p<0.01, ***p<0.001 compared with model (35-day-old female *Drosophila*); ^###^ *p*<0.001 compared with control (7-day-old female *Drosophila*).

Ovarian size was dominated by age. The length and diameter of older ovaries were shortened and atrophied. Ginsenosides maintained the size (length and diameter) of the aging ovary to prevent the atrophy, with a dose-dependent pattern (**Figures 2A–C**). Ovarian atrophy leads to a decrease in steroid hormones secretion, reducing ovarian function. Levels of steroid hormones, including 20E and JH, were consumed with age. The levels of 20E and JH in aged female *Drosophila* treated with ginsenoside were significantly increased (**Figures 2D, E**). These results suggested that ginsenoside treatment delay ovarian dysfunction of aged female *Drosophila*.

### Effects of ginsenosides on steroid signaling

**Figure 3:**
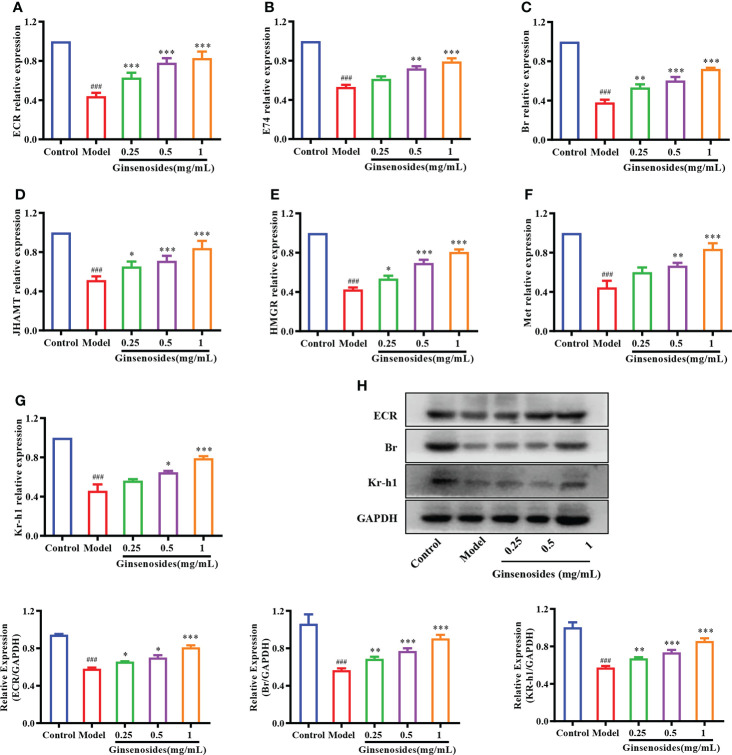
Effect of ginsenosides on expression of steroidal signal-related genes in *Drosophila*. **(A)** ECR; **(B)** E74; **(C)** Br; **(D)** JHAMT; **(E)** HMGR; **(F)** Met; **(G)** Kr-h1; **(H)** Relative expression levels of ECR, Br and Kr-h1; GAPDH antibody was used as loading control. ^###^ *p*<0.001 compared with control (7-day-old female *Drosophila*); *p<0.05, **p<0.01, ***p<0.001 compared with model (35-day-old female *Drosophila*).

To determine whether ginsenosides enhanced reproductive capacity through triggering of steroid signals, we measured expression of genes related to steroid signaling pathways in aged female *Drosophila*. Expression levels of 20E receptor genes encoding proteins ECR, early transcription factor E74 and Br were reduced in model group as compared with levels in control group, but were all significantly improved with ginsenoside treatment (**Figures 3A–C**). In addition, 20E could promote the expression of juvenile hormone acid O-methyltransferase (JHAMT) and Hydroxyl-methylglutaryl-CoA reductase (HMGR) genes, thus promoting JH biosynthesis (34). The expression levels of JHAMT and HMGR in the model group were lower than those in the control group, and increased after ginsenosides treatment (**Figures 3D, E**). Meanwhile, expression levels of Kruppel Homolog 1 (Kr-h1) and Methoprene Tolerant (Met) increased in a dose-dependent manner with increasing ginsenosides concentration (**Figures 3F, G**). Again, we verified that the protein expression levels of 20E receptor protein ECR, early transcription factor Br and Kr-h1 protein were reduced in the model group compared with the control group, but all were significantly increased by ginsenosides treatment, in line with mRNA expression (**Figure 3H**). These results indicate that ginsenosides activated steroid signaling to promote the reproductive capacity of aged female *Drosophila*.

### ECR dependence of ginsenosides to improve reproductive ability

**Figure 4:**
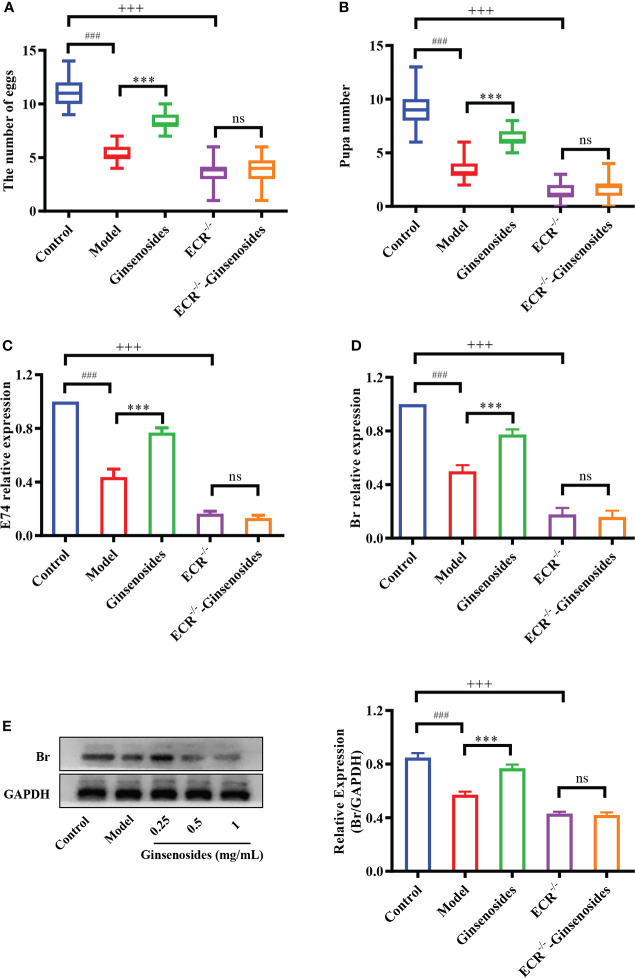
Effect of ginsenosides on the reproductive capacity of ECR mutant *Drosophila*. **(A)** The number of eggs; **(B)** The number of pupae; **(C)** Relative expression of gene encoding E74; **(D)** Relative expression of Br; **(E)** Relative expression levels of Br; GAPDH antibody was used as loading control. ^###^ *p*<0.001 model compared with control (7-day-old female *Drosophila*); ***p<0.001 compared with model (35-day-old female *Drosophila*); ^+++^ *p*<0.001 ECR^-/-^ compared with control (35-day-old female *Drosophila*); ns, no significance compared with ECR^-/-^.

Ginsenosides activated steroid signaling was verified in ECR mutant female *Drosophila*. Older *Drosophila* had reduced reproductive capacity compared to younger *Drosophila*, and ginsenosides could increase the numbers of egg and pupal in older females. In addition, the reproductive ability of ECR mutant *Drosophila* was reduced compared to normal *Drosophila* and was not improved by feeding ginsenosides (**Figures 4A, B**). This indicates that ginsenosides depend on ECR to enhance the reproductive ability of older female *Drosophila*. Moreover, ginsenosides had no effect on the expression of steroidal signaling related genes E74 and Br in ECR knockdown mutant *Drosophila* (**Figures 4C, D**). Additionally, in the ECR knockout mutant *Drosophila*, ginsenosides exhibited no impact on the expression of the sterol signaling-related protein Br **(** **Figure 4E** **)**. That is to say, ginsenosides were dependent on ECR to activate steroid signals and improve reproductive capacity.

### Screening of ginsenosides monomer with reproductive promotion

**Figure 5:**
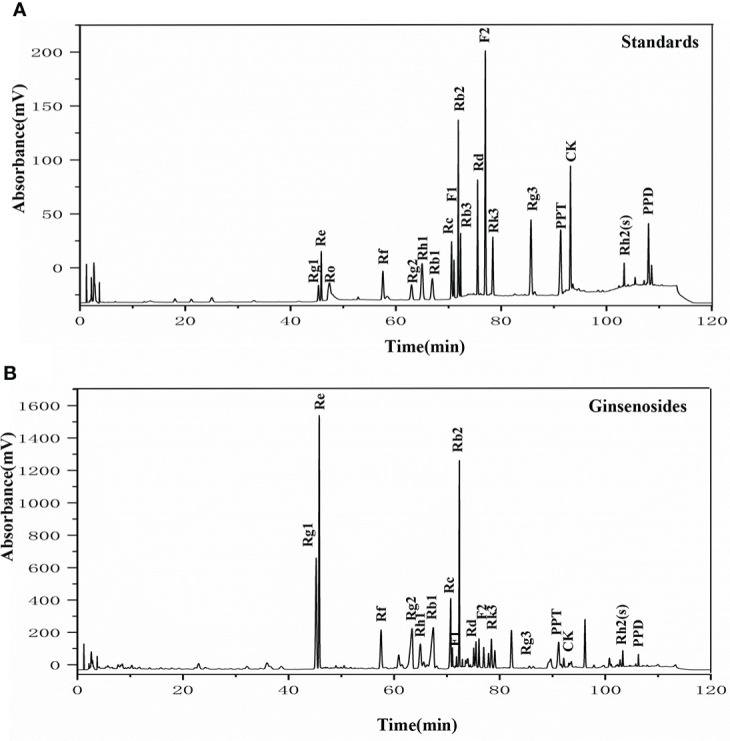
The HPLC chromatogram of ginsenosides. **(A)** HPLC analysis of the standard ginsenosides monomers including Rg1, Re, Ro, Rf, Rg2, Rh1, Rb1, Rc, F1, Rb2, Rb3, Rd, F2, Rk3, Rg3, PPT, CK, Rh2(s), and PPD; **(B)** HPLC chromatogram of ginsenosides test samples, with UV detection at 203 nm.

**Figure 6:**
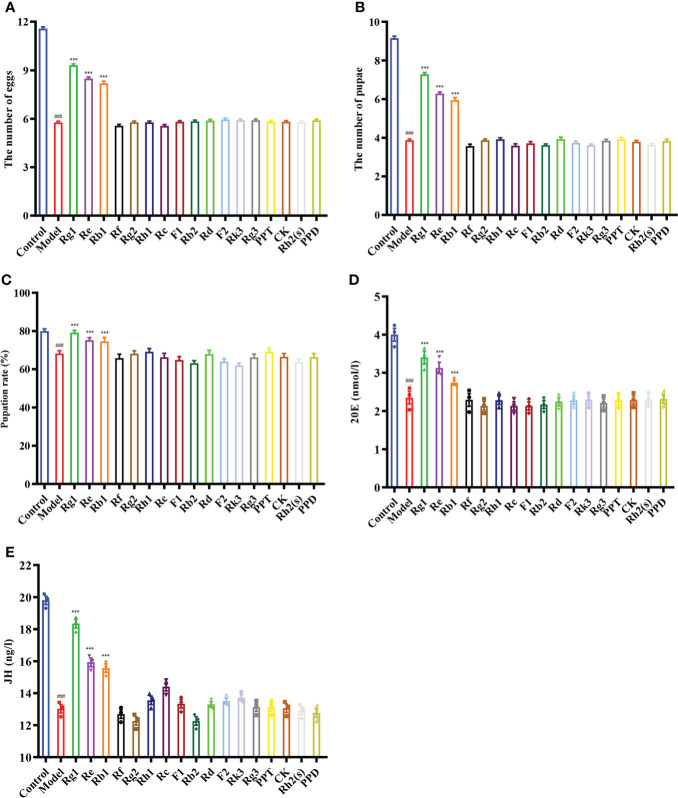
Effect of ginsenoside monomers on the reproductive ability and steroid hormone levels of *Drosophila*. **(A)** The number of eggs; **(B)** The number of pupae; **(C)** Pupation rate; **(D)** 20E content; **(E)** JH content; Rg1, 0.26 mg/mL; Re, 0.19 mg/mL; Rg2, 0.13 mg/mL; Rb1, 0.06 mg/mL; Rf, 0.03 mg/mL; Rh2(s), 0.02 mg/mL; Rh1, 0.02 mg/mL; PPT, 0.01 mg/mL; Rk3, 0.01 mg/mL; Rd, 0.007 mg/mL; Rc, 0.006 mg/mL; F2, 0.004 mg/mL; Rb2, 0.004 mg/mL; CK, 0.003 mg/mL; F1, 0.003 mg/mL; PPD, 0.002 mg/mL; Rg3, 0.001 mg/mL; according to the concentration ratio of ginsenosides at 1 mg/mL. ^###^ *p*<0.001 compared with control (7-day-old female *Drosophila*); ***p<0.001 compared with model (35-day-old female *Drosophila*).

To elucidate the components of the ginsenosides that might be responsible for improving reproductive effects, ginsenoside monomers were identified and quantified from the total ginsenosides by HPLC. As shown in **Figure 5**, ginsenoside monomers were included Rg1 (26.41%), Re (18.61%), Rg2 (12.54%), Rb3 (8.19%), Rb1 (6.10%), Rf (3.18%), Rh2S (1.84%), Rh1 (1.77%), PPT (1.48%), Rk3 (1.40%), Rd (0.71%), Rc (0.56%), F2 (0.39%), Rb2 (0.35%), CK (0.32%), F1 (0.29%), PPD (0.24%), and Rg3 (0.12%), with the concentration of 84.52%, according to the standard curve of the different ginsenosides. Subsequently, the effects of 17 ginsenoside monomers on reproductive capacity and steroid hormone levels of 35-day-old female *Drosophila* were analyzed. Rg1, Rb1 and Re were significantly increased the number of eggs by 1.6, 1.5 and 1.4 times, respectively. Meanwhile, Rg1, Rb1 and Re were also increased the number and pupation rate of pupae. The other 14 ginsenosides monomers had no significant effect (**Figures 6A–C**). In addition, Rg1, Rb1 and Re also promoted steroid hormone secretion, and the increase of steroid hormone content was more significant in older female *Drosophila* (**Figures 6D, E**). These results suggested that Rg1, Rb1 and Re might be the active components of total ginsenosides to improve the reproductive capacity of elderly female *Drosophila*.

## Discussion

After the age of 30, the female enters the decline period of ovarian function, which is the fundamental cause of a series of aging phenomena. Delaying ovarian senescence can essentially solve age-related fertility disorders and prolong healthy life. Ginseng is viewed as a healthy energy tonic which can delay aging and prolong life (35). It has been shown that ginsenosides increased the viability of older female *Drosophila* (36). Furthermore, it has been reported that ginsenosides can prolong the estrus cycle of mature females and increase the egg-laying capacity of queen bees (37). Ginsenosides being added to the culture medium encouraged the growth of chicken ovarian germ cells *in vitro* (38). *In vitro* experiments showed that ginsenosides could enhance follicular cell development in mice (39). In our study, the reproductive ability of aged female *Drosophila* was significantly lower than that of young females. Ginsenosides could increase the number of eggs and pupae in aged females. It suggests that ginsenosides improved the quantity and quality of the offspring, prolonged life and restored muscle ability to the young state in aged female *Drosophila*, thus played the role of protecting the reproductive and delaying the aging.

**Figure 7:**
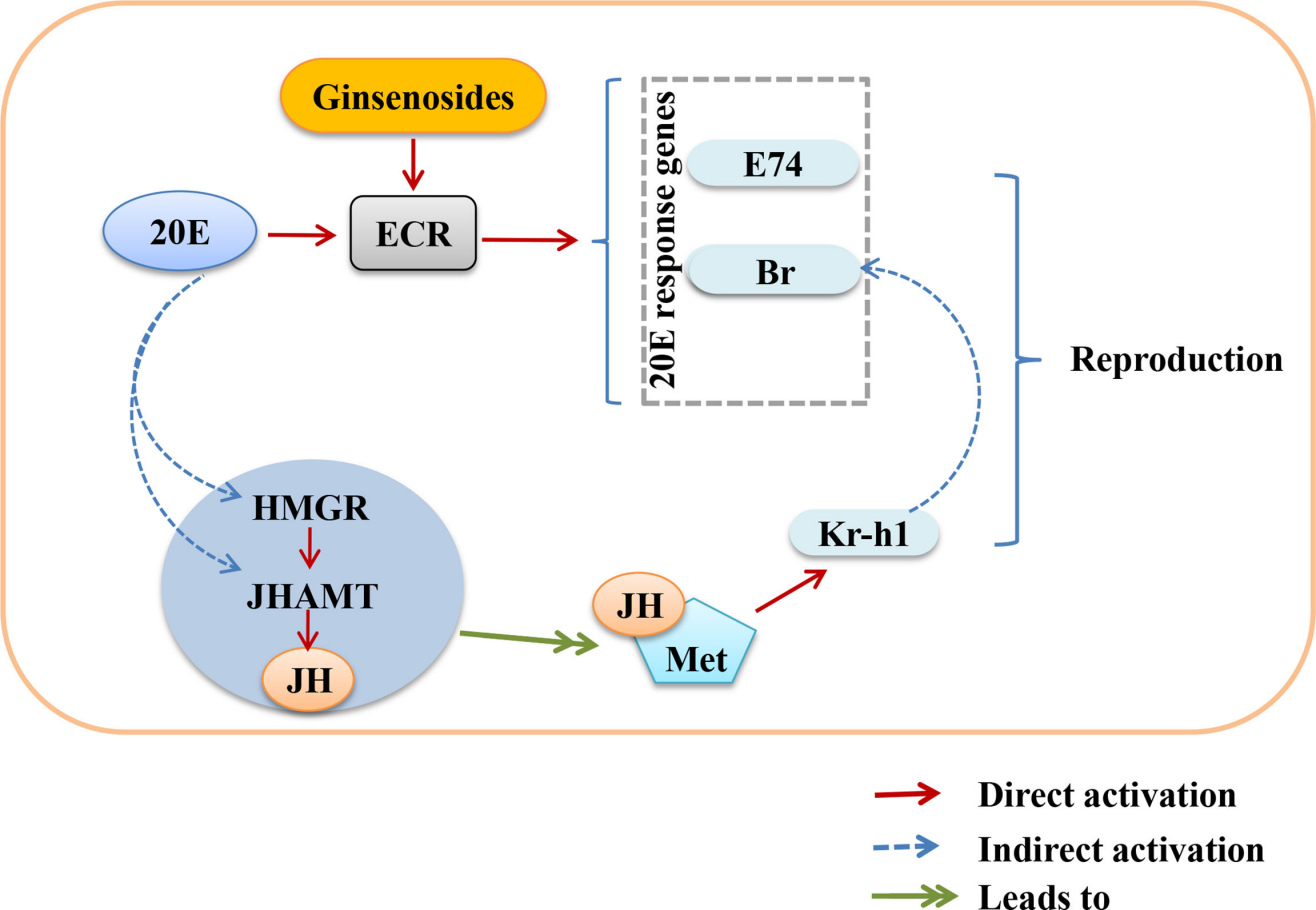
Overview showing the relationship between activation of nuclear receptor ECR by ginsenosides turns on steroid hormone signaling to improve reproductive capacity in aged female *Drosophila*.

Ovarian function deteriorates as women age, resulting in a lack of steroid hormones and infertility (40, 41). Ginsenosides can promote the release of steroid hormones to play an estrogen-like role (42). During the larval stage, 20E starts to induce larval pupation, while JH keeps *Drosophila* in the immature state by inhibiting the metamorphosis of 20E, thus ensuring that the metamorphosis time is reasonable. In the adult stage, the decrease of 20E or JH content in the ovary also reduces the reproductive capacity. 20E and JH promote reproduction through multiple pathways, such as oogenesis, oocyte formation and chorionic vitellogenesis (43, 44). The ovarian size was dominated by age. The length and diameter of older ovaries were shortened and atrophied. We found that ginsenosides maintained the size of the aging ovary to prevent atrophy, with a dose-dependent pattern. Ovarian atrophy leads to a decrease in steroid hormones secretion, reducing ovarian function. Levels of steroid hormones, including 20E and JH, were consumed with age. The levels of 20E and JH in aged female *Drosophila* treated with ginsenosides were significantly increased. Our results further demonstrate that ginsenosides treatment delay ovarian dysfunction of aged female *Drosophila*. 20E has been reported to affect gonadal development and oogenesis by regulating mitosis and meiosis (44). In adult *Drosophila*, 20E affects courtship behavior, yolk production and egg laying, reproductive lag, innate immunity, and resistance and longevity (34, 45). Several studies have shown that ginsenosides by binding the intracellular nuclear hormone receptors, such as the proliferator-activated receptor, androgen receptor (AR), estrogen receptor (ER), and progesterone receptor (PR), can activate the genomic pathway (38). 20E acts through a heterodimeric receptor consisting of ECR and ultraspiracle protein (USP), leading to transcriptional activation of early 20E-inducible genes, including the downstream genes E74 and Br (46). 20E is similar to human estrogen, which also indicates that ginsenosides have estrogen-like effects that bind to their receptors to perform their functions. In this study, ginsenosides promoted the expression of ECR and induced the transcription of downstream genes E74 and Br to activate 20E signaling. Moreover, 20E promoted JH biosynthesis by promoting the expression of HMGR and JHAMT, thus increasing the level of JH. Studies had shown that reproductive capacity could be maintained by regulating the expression of JHAMT and HMGR (47). JH could bind to Met receptor to form an active complex that promoted the transcription of response genes Kr-h1 and Br and facilitates oogenesis (48). We found that 20E promoted JH biosynthesis by enhancing HMGR and JHAMT expression. Ginsenosides enhanced the synergistic effect of JH signal and 20E signal to promote the reproductive capacity of aged female *Drosophila.* However, alterations in ECR function can affect the formation of mating memory and the selection of courtship partners in *Drosophila* (49). In our study, ginsenosides did not improve steroid signaling-related genes and reproductive capacity in female *Drosophila* with ECR knockdown. These results suggested that ginsenosides depended on the activation of nuclear receptor ECR to turn on steroid signaling pathway, thus improving the fertility of older female *Drosophila* (**Figure 7**).

There are about 40 ginsenoside monomers with definite structures, all of which contain sterane steroid nuclei arranged in four rings by 30 carbon atoms. The multiple pharmacological activities of various ginsenoside monomers have been reported, but there are relatively few studies on improving the fertility decline caused by aging. To excavate the ginsenoside monomers that might be responsible for improving reproductive effects, various monomers were identified and quantified by HPLC. It was found that Rg1, Re, and Rb1 exhibited similar effects as the total ginsenosides. Ginsenoside Rg1 has been shown to improve fertility and repair ovarian damage in a mouse with premature ovarian failure (11). Meanwhile, ginsenoside Rb1 has been shown to protect porcine oocytes from damage, thus improving parthenogenesis and embryo quality after *in vitro* fertilization (50). These studies are consistent with our conclusions. However, individual treatments with Rg1, Re, and Rb1 were less effective than that of ginsenosides. Therefore, it was speculated that there were synergistic and synergistic effects among various ginsenosides monomers, which should be the subject of further research.

In conclusion, ginsenosides depend on ECR to activate steroid signals to improve the reproductive capacity of aged female *Drosophila*, which play an estrogen-like role. It is noteworthy that this effect may be commonly performed by ginsenoside monomers such as Rg1, Re and Rb1. This study provides new insights for the further development of ginsenosides as active components for alleviating female reproductive aging. However, multiple ginsenoside monomers may exert synergistic effect through different drug therapy approaches. We will continue to investigate in depth the complex interactions between different ginsenosides naturally present in ginseng roots. This will provide an experimental basis for the development of natural drugs for the treatment and prevention of diseases related to reproductive aging to benefit more women with fertility needs.

## Data availability statement

The raw data supporting the conclusions of this article will be made available by the authors, without undue reservation.

## Ethics statement

The animal study was reviewed and approved by the Ethics Committee of Changchun University of Chinese Medicine of TCM (No: 2021349).

## Author contributions

BF and RM designed the study and drafted, revised the manuscript, conducted experiments, extracted and analyzed the experimental data. BF, FL, and XC performed the experiments. XT, PY, and JL analyzed the data and performed the statistical analysis. DZ and LS evaluated the quality of the whole study and drafted the manuscript. RM and LS provided funding support. All authors contributed to the article and approved the submitted version.

## Funding

This work was supported by the National Natural Science Foundation of China (No. U20A20402) and the Science and Technology Development of Jilin Province (No. 20210304002YY and YDZJ202201ZYTS167).

## Conflict of interest

The authors declare that the research was conducted in the absence of any commercial or financial relationships that could be construed as a potential conflict of interest.

## Publisher’s note

All claims expressed in this article are solely those of the authors and do not necessarily represent those of their affiliated organizations, or those of the publisher, the editors and the reviewers. Any product that may be evaluated in this article, or claim that may be made by its manufacturer, is not guaranteed or endorsed by the publisher.

## References

[1] Schwartz K Martin C Hipp H Kawwass J. Pregnancy and fertility concerns: A survey of United States obstetrics and gynecology residents. Matern Child Hlth J (2021) 25(1):172–9. doi: 10.1007/s10995-020-03027-w 33242208

[2] Carson S Kallen A. Diagnosis and management of infertility: A review. JAMA (2021) 326(1):65–76. doi: 10.1001/jama.2021.4788 34228062 PMC9302705

[3] Li C Lin L Tsai H Chern C Wen Z Wang P. The molecular regulation in the pathophysiology in ovarian aging. Aging Dis (2021) 12(3):934–49. doi: 10.14336/ad.2020.1113 PMC8139203 34094652

[4] Zu Y Yang J Zhang C Liu D. The pathological mechanisms of estrogen-induced cholestasis: Current perspectives. Front Pharmacol (2021) 12:761255. doi: 10.3389/fphar.2021.761255 34819862 PMC8606790

[5] Kumar G Du B Chen J. Effects and mechanisms of dietary bioactive compounds on breast cancer prevention. Pharmacol Res (2021) 178:105974. doi: 10.1016/j.phrs.2021.105974 34818569

[6] Mastorakos G Iatrakis G Zervoudis S Syropoulou S. Progestins and the risk of breast cancer. Acta Endocrinol-Buch (2021) 17(1):90–100. doi: 10.4183/aeb.2021.90 PMC8417494 34539915

[7] Ghamari K Kashani L Jafarinia M Tadayon Najafabadi B Shokraee K Esalatmanesh S. Vitamin e and ginseng supplementation to enhance female sexual function: A randomized, double-blind, placebo-controlled, clinical trial. Women Health (2020) 60(10):1164–73. doi: 10.1080/03630242.2020.1803465 32893745

[8] Ma S Ma R Xia T Afnan M Song X Xu F. Efficacy and safety of ding-Kun-Dan for female infertility patients with predicted poor ovarian response undergoing *in vitro* fertilization/intracytoplasmic sperm injection: Study protocol for a randomized controlled trial. Trials (2018) 19(1):124. doi: 10.1186/s13063-018-2511-0 29458401 PMC5819272

[9] Lee JH Choi SH Kwon OS Shin TJ Lee JH Lee BH. Effects of ginsenosides, active ingredients of panax ginseng, on development, growth, and life span of caenorhabditis elegans. Biol Pharm Bull (2007) 30(11):2126–34. doi: 10.1248/bpb.30.2126 17978487

[10] He L Ling L Wei T Wang Y Xiong Z. Ginsenoside Rg1 improves fertility and reduces ovarian pathological damages in premature ovarian failure model of mice. Exp Biol Med (2017) 242(7):683–91. doi: 10.1177/1535370217693323 PMC5363692 28178855

[11] Xu X Qu Z Qian H Li Z Sun X Zhao X. Ginsenoside Rg1 ameliorates reproductive function injury in C57BL/6J mice induced by di-n-butyl-phthalate. Environ Toxicol (2021) 36(5):789–99. doi: 10.1002/tox.23081 33331133

[12] Lee JH Ahn JY Shin TJ Choi SH Lee BH Hwang SH. Effects of minor ginsenosides, ginsenoside metabolites, and ginsenoside epimers on the growth of caenorhabditis elegans. J Ginseng Res (2011) 35(3):375–83. doi: 10.5142/jgr.2011.35.3.375 PMC3659541 23717083

[13] Jin W Ma R Zhai L Xu X Lou T Huang Q. Ginsenoside Rd attenuates ACTH-induced corticosterone secretion by blocking the MC2R-cAMP/PKA/CREB pathway in Y1 mouse adrenocortical cells. Life Sci (2020) 245:117337. doi: 10.1016/j.lfs.2020.117337 31972205

[14] Du J Cheng B Zhu X Ling C. Ginsenoside Rg1, a novel glucocorticoid receptor agonist of plant origin, maintains glucocorticoid efficacy with reduced side effects. J Immunol (2011) 187(2):942–50. doi: 10.4049/jimmunol.1002579 21666059

[15] Hao K Gong P Sun S Hao H Wang G Dai Y. Beneficial estrogen-like effects of ginsenoside Rb1, an active component of panax ginseng, on neural 5-HT disposition and behavioral tasks in ovariectomized mice. Eur J Pharmacol (2011) 659(1):15–25. doi: 10.1016/j.ejphar.2011.03.005 21414307

[16] Colella M Cuomo D Peluso T Falanga I Mallardo M De Felice M. Ovarian aging: Role of pituitary-ovarian axis hormones and ncRNAs in regulating ovarian mitochondrial activity. Front Endocrinol (Lausanne) (2021) 12:791071. doi: 10.3389/fendo.2021.791071 34975760 PMC8716494

[17] Ikeda K Horie-Inoue K Inoue S. Functions of estrogen and estrogen receptor signaling on skeletal muscle. J Steroid Biochem Mol Biol (2019) 191:105375. doi: 10.1016/j.jsbmb.2019.105375 31067490

[18] Lin XM Chen M Wang QL Ye XM Chen HF. Clinical observation of kuntai capsule combined with fenmotong in treatment of decline of ovarian reserve function. World J Clin cases (2021) 9(28):8349–57. doi: 10.12998/wjcc.v9.i28.8349 PMC8554447 34754844

[19] Ahmed S Maldera J Krunic D Paiva-Silva G Pénalva C Teleman A. Fitness trade-offs incurred by ovary-to-gut steroid signalling in *Drosophila* . Nature (2020) 584(7821):415–9. doi: 10.1038/s41586-020-2462-y PMC7442704 32641829

[20] Sharma V Pandey A Kumar A Misra S Gupta H Gupta S. Functional male accessory glands and fertility in *Drosophila* require novel ecdysone receptor. PloS Genet (2017) 13(5):e1006788. doi: 10.1371/journal.pgen.1006788 28493870 PMC5444863

[21] Luo W Liu S Zhang W Yang L Huang J Zhou S. Juvenile hormone signaling promotes ovulation and maintains egg shape by inducing expression of extracellular matrix genes. P Natl Acad Sci USA (2021) 118(39):2104461118. doi: 10.1073/pnas.2104461118 PMC8488625 34544864

[22] Khalid M Ahmad S Ngegba P Zhong G. Role of endocrine system in the regulation of female insect reproduction. Biology (2021) 10(7):614. doi: 10.3390/biology10070614 34356469 PMC8301000

[23] Hou W Pei J. *Drosophila* melanogasterProteomic analysis of red ginseng on prolonging the life span of Male. Front Pharmacol (2021) 12:618123. doi: 10.3389/fphar.2021.618123 34177563 PMC8232884

[24] Beachum A Whitehead K McDonald S Phipps D Berghout H Ables E. Orphan nuclear receptor ftz-f1 (NR5A3) promotes egg chamber survival in the *Drosophila* ovary. G3 (Bethesda) (2021) 11(2):kab003. doi: 10.1093/g3journal/jkab003 PMC8022936 33693603

[25] Mukherjee N Mukherjee C. Germ cell ribonucleoprotein granules in different clades of life: From insects to mammals. Wirs RNA RNA (2021) 12(4):e1642. doi: 10.1002/wrna.1642 33555143

[26] Teseo S Houot B Yang K Monnier V Liu G Tricoire HG. Sinense and extracts improve healthspan of aging flies and provide protection in a huntington disease model. Aging Dis (2021) 12(2):425–40. doi: 10.14336/ad.2020.0714-1 PMC7990376 33815875

[27] Moadeli T Mainali B Ponton F Taylor PW. Effects of fatty acids and vitamin e in larval diets on development and performance of Queensland fruit fly. J Insect Physiol (2020) 125:104058. doi: 10.1016/j.jinsphys.2020.104058 32422147

[28] Beghelli D Zallocco L Barbalace MC Paglia S Strocchi S Cirilli I. Pterostilbene promotes mean lifespan in both Male and female *Drosophila* melanogaster modulating different proteins in the two sexes. Oxid Med Cell Longev (2022) 2022:1744408. doi: 10.1155/2022/1744408 35222791 PMC8865974

[29] Baenas N Wagner AE. *Drosophila* melanogaster as a model organism for obesity and type-2 diabetes mellitus by applying high-sugar and high-fat diets. Biomolecules (2022) 12(2):307. doi: 10.3390/biom12020307 35204807 PMC8869196

[30] Lu J Huang Q Zhang D Lan T Zhang Y Tang X. The protective effect of DiDang tang against AlCl3-induced oxidative stress and apoptosis in PC12 cells through the activation of SIRT1-mediated Akt/Nrf2/HO-1 pathway. Front Pharmacol (2020) 11:466. doi: 10.3389/fphar.2020.00466 32372957 PMC7179660

[31] Zhai L Xu X Liu J Jing C Yang X Zhao D. A novel biochemical study of anti-dermal fibroblast replicative senescence potential of panax notoginseng oligosaccharides. Front Pharmacol (2021) 12:690538. doi: 10.3389/fphar.2021.690538 34276377 PMC8277921

[32] Li Z Jiang R Wang M Zhai L Liu J Xu X. Ginsenosides repair UVB-induced skin barrier damage in BALB/c hairless mice and HaCaT keratinocytes. J Ginseng Res (2022) 46(1):115–25. doi: 10.1016/j.jgr.2021.05.001 PMC8753432 35035244

[33] Wang H Zhang S Zhai L Sun L Zhao D Wang Z. Ginsenoside extract from ginseng extends lifespan and health span in caenorhabditis elegans. Food Funct (2021) 12(15):6793–808. doi: 10.1039/d1fo00576f 34109970

[34] Song J Zhou S. Post-transcriptional regulation of insect metamorphosis and oogenesis. Cell Mol Life Sci (2020) 77(10):1893–909. doi: 10.1007/s00018-019-03361-5 PMC11105025 31724082

[35] Yu X Li H Lin D Guo W Xu Z Wang L. Ginsenoside prolongs the lifespan of c. elegans *via* lipid metabolism and activating the stress response signaling pathway. Int J Mol Sci (2021) 22(18):9668. doi: 10.3390/ijms22189668 34575832 PMC8465798

[36] Liu QX Zhang W Wang J Hou W Wang YP. A proteomic approach reveals the differential protein expression in *Drosophila* melanogaster treated with red ginseng extract (Panax ginseng). J Ginseng Res (2018) 42(3):343–51. doi: 10.1016/j.jgr.2017.04.006 PMC6026366 29983616

[37] Lin H Liu Z Pi Z Men L Chen W Liu Z. Urinary metabolomic study of the antagonistic effect of p. ginseng in rats with estrogen decline using ultra performance liquid chromatography coupled with quadrupole time-of-flight mass spectrometry. Food Funct (2018) 9(3):1444–53. doi: 10.1039/c7fo01680h 29457805

[38] Park J Song H Kim SK Lee MS Rhee DK Lee Y. Effects of ginseng on two main sex steroid hormone receptors: Estrogen and androgen receptors. J Ginseng Res (2017) 41(2):215–21. doi: 10.1016/j.jgr.2016.08.005 PMC5386121 28413327

[39] Majdi Seghinsara A Shoorei H Hassanzadeh Taheri MM Khaki A Shokoohi M Tahmasebi M. Panax ginseng extract improves follicular development after mouse preantral follicle 3D culture. Cell J (2019) 21(2):210–9. doi: 10.22074/cellj.2019.5733 PMC6397605 30825295

[40] Gurvich C Le J Thomas N Thomas E Kulkarni J. Sex hormones and cognition in aging. Vitam Horm (2021) 115:511–33. doi: 10.1016/bs.vh.2020.12.020 33706960

[41] Björkgren I Chung D Mendoza S Gabelev-Khasin L Petersen N Modzelewski A. Alpha/Beta hydrolase domain-containing protein 2 regulates the rhythm of follicular maturation and estrous stages of the female reproductive cycle. Front Cell Dev Biol (2021) 9:710864. doi: 10.3389/fcell.2021.710864 34568325 PMC8455887

[42] Tian M Li L Zheng R Yang L Wang Z. Advances on hormone-like activity of panax ginseng and ginsenosides. Chin J Nat Medicines (2020) 18(7):526–35. doi: 10.1016/s1875-5364(20)30063-7 32616193

[43] Al Baki M Lee D Jung J Kim Y. Insulin signaling mediates previtellogenic development and enhances juvenile hormone-mediated vitellogenesis in a lepidopteran insect, maruca vitrata. BMC Dev Bio (2019) 19(1):14. doi: 10.1186/s12861-019-0194-8 31277577 PMC6610926

[44] Zhu Z Tong C Qiu B Yang H Xu J Zheng S. 20E-mediated regulation of BmKr-h1 by BmKRP promotes oocyte maturation. BMC Biol (2021) 19(1):39. doi: 10.1186/s12915-021-00952-2 33632227 PMC7905918

[45] Tricoire H Battisti V Trannoy S Lasbleiz C Pret AM Monnier V. The steroid hormone receptor EcR finely modulates *drosophila* lifespan during adulthood in a sex-specific manner. Mech Ageing Dev (2009) 130(8):547–52. doi: 10.1016/j.mad.2009.05.004 19486910

[46] Uyehara CM McKay DJ. Direct and widespread role for the nuclear receptor EcR in mediating the response to ecdysone in *drosophila* . Proc Natl Acad Sci USA (2019) 116(20):9893–902. doi: 10.1073/pnas.1900343116 PMC6525475 31019084

[47] Sheng Z Ma L Cao M Jiang R Li S. Juvenile hormone acid methyl transferase is a key regulatory enzyme for juvenile hormone synthesis in the eri silkworm, samia cynthica ricini. Arch Insect Biochem (2008) 69(3):143–54. doi: 10.1002/arch.20268 18839418

[48] Gu S Chen C Lin P. Changes in expressions of ecdysteroidogenic enzyme and ecdysteroid signaling genes in relation to bombyx embryonic development. J Exp Zool Part A (2021) 335(5):477–88. doi: 10.1002/jez.2466 33929096

[49] Carney GE Bender M. The *drosophila* ecdysone receptor (EcR) gene is required maternally for normal oogenesis. Genetics (2000) 154(3):1203–11. doi: 10.1093/genetics/154.3.1203 PMC1461007 10757764

[50] Luo Z Xuan M Han S Li Z Khan N Quan B. Ginsenoside Rb1 protects porcine oocytes against methylglyoxal damage thus it improves the quality of parthenogenetic activation and *in vitro* fertilization embryos. P Natl Acad Sci USA (2021) 36(4):586–97. doi: 10.1002/tox.23063 33236476

